# Supplementary material for: Specialized curricula for training vision language models in retinal image analysis
Source: NPJ Digit Med. 2025 Aug 19;8:532. doi: 10.1038/s41746-025-01893-8 (PMC12365215; doi:10.1038/s41746-025-01893-8)
Supplement: Supplementary file 1 — Supplementary Information [file 41746_2025_1893_MOESM1_ESM.pdf]

A Supplementary material

A.1 Supporting methodological and results figures

a

Anatomical	Count	Mean and IQR
Eye side (left)	22,701	--
Eye side (right)	19,225	--
**Demographic**	**Count**	**Mean and IQR**
Patient Sex (Female)	25,825	--
Patient Sex (Male)	16,101	--
Patient age in years	41,926	81.8 (77.0, 88.0)
**Image quality**	**Count**	**Mean and IQR**
Image quality index	41,926	20.6 (17.7, 23.2)
**Biomarker and disease related**	**Count**	**Mean and IQR**
EHR disease stage	38,123	--
Visual acuity in Letter score	34,599	58.0 (44.0, 75.0)
Hypertransmission	12,653	--
iRORA	6,500	--
Healthy (no AMD)	5,897	--
RPE elevation	4,986	--
Tiny drusen	3,190	--
cRORA	3,023	--
Macular scar	2,644	--
PED	2,463	--
Grainy image quality	2,275	--
Small RPE elevation	1,811	--
Disciform scar	1,520	--
Fibrovascular PED	1,508	--
Scan not centered on fovea	1,374	--
Poor image quality	1,284	--
Large drusen	1,283	--
Poor image contrast	1,269	--
Drusenoid PED	1,237	--
Double-layer sign	1,212	--
Optical disc	1,073	--
Deep foveal pit	982	--
Medium drusen	977	--
Subretinal fluid	969	--
Intraretinal fluid	885	--
Vitreomacular interface abnormalities	849	--
Small intraretinal fluid	762	--
Large subretinal fluid	410	--

b

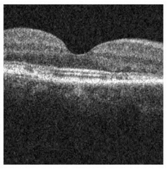

Tabular report:
- Imaging biomarkers visible in the image: **no visible AMD biomarkers**
- Imaging biomarkers not present/detected: **pigment epithelial detachment, hypertransmission and subretinal hyperreflective material**
- Subject's age: **66**
- Sex of the patient: **male**
- Image quality rating: **ok**
- Eye scanned: **right**

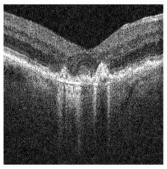

Tabular report:
- Imaging biomarkers visible in the image: **iRORA**
- Imaging biomarkers not present/detected: **cRORA, double-layer sign and subretinal hyperreflective material**
- AMD stage of the patient: **late wet AMD**
- Visual acuity (measured in letter score): **62**
- Subject's age: **79**
- Sex of the patient: **female**
- Image quality rating: **ok**
- Eye scanned: **right**

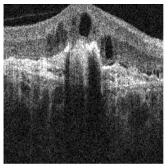

Tabular report:
- Imaging biomarkers visible in the image: **pigment epithelial detachment, subretinal hyperreflective material and hypertransmission**
- Imaging biomarkers not present/detected: **cRORA, small drusen and tiny drusen**
- Visual acuity (measured in letter score): **16**
- Subject's age: **69**
- Sex of the patient: **male**
- Image quality rating: **good**
- Eye scanned: **right**

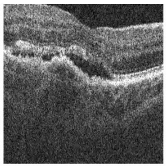

Tabular report:
- Imaging biomarkers visible in the image: **subretinal fluid and pigment epithelial detachment**
- Imaging biomarkers not present/detected: **large drusen, hypertransmission and iRORA**
- AMD stage of the patient: **late wet AMD**
- Visual acuity (measured in letter score): **69**
- Subject's age: **74**
- Sex of the patient: **female**
- Image quality rating: **very poor**
- Eye scanned: **right**

Supplementary Figure 1: Summary statistics and tabular reports for data used to create curriculum part 1. (**a**) The count of each tabular label used to create the tabular reports for the training portion of curriculum part 1. Labels are divided into those relating to anatomy, demography, quality, and biomarkers and disease. By default, variables are not mutually exclusive (an image may show drusen and subretinal fluid), with the exception of those introduced with a bracketed notation, such as Eye side (left) and Eye side (right). We report mean and inter-quartile bounds for continuous-valued variables, and leave this blank for categorical variables (**b**) Four randomly selected images and their corresponding tabular reports in text form.

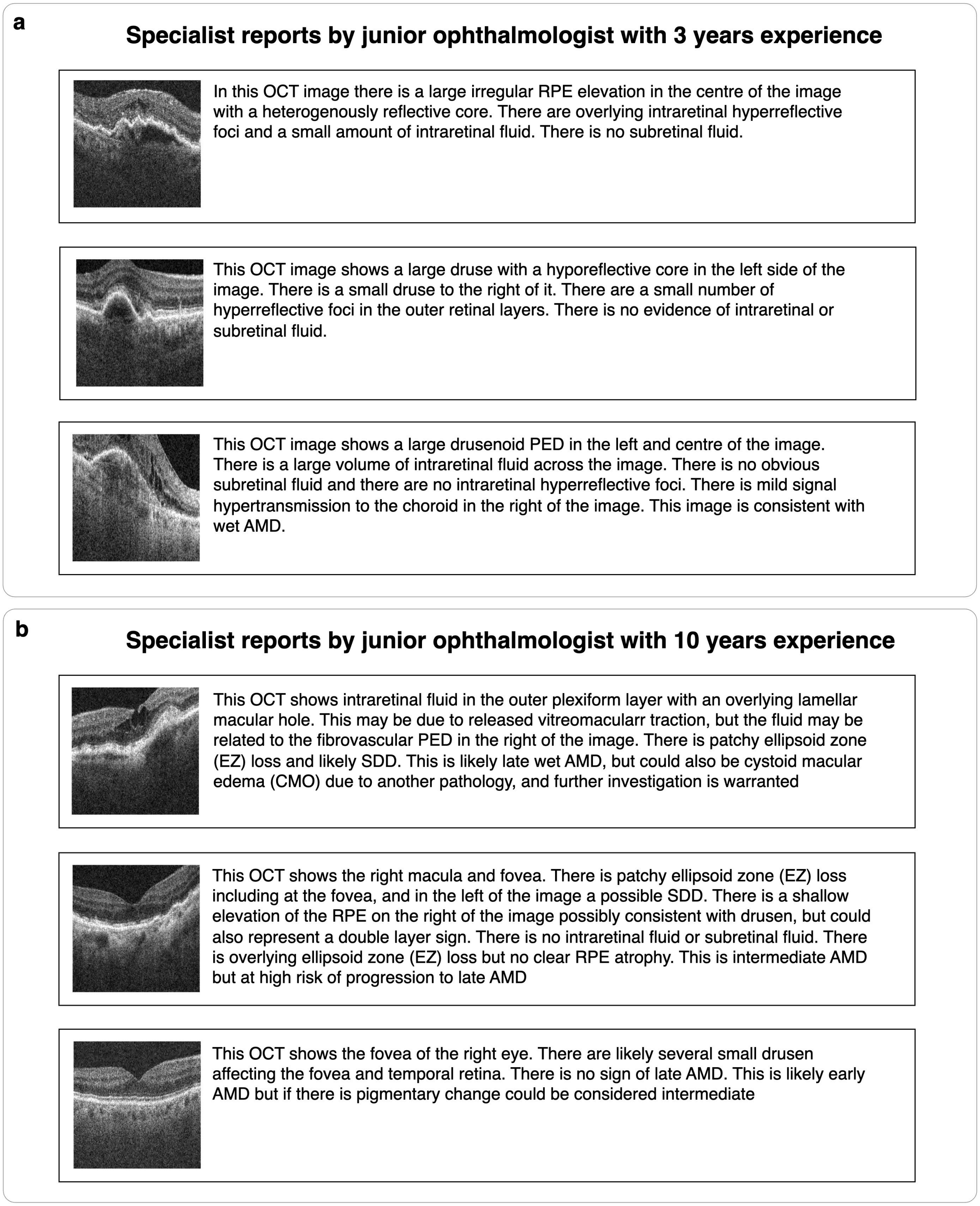

Supplementary Figure 2: Sample textual reports curated by ophthalmologists used to create curriculum part 2. (**a**) Three example reports randomly selected from the 244 written by the first junior ophthalmologist with 3 years experience in ophthalmology. (**b**) Three example reports randomly selected from the 86 written by the more experienced junior ophthalmologist with 10 years experience.

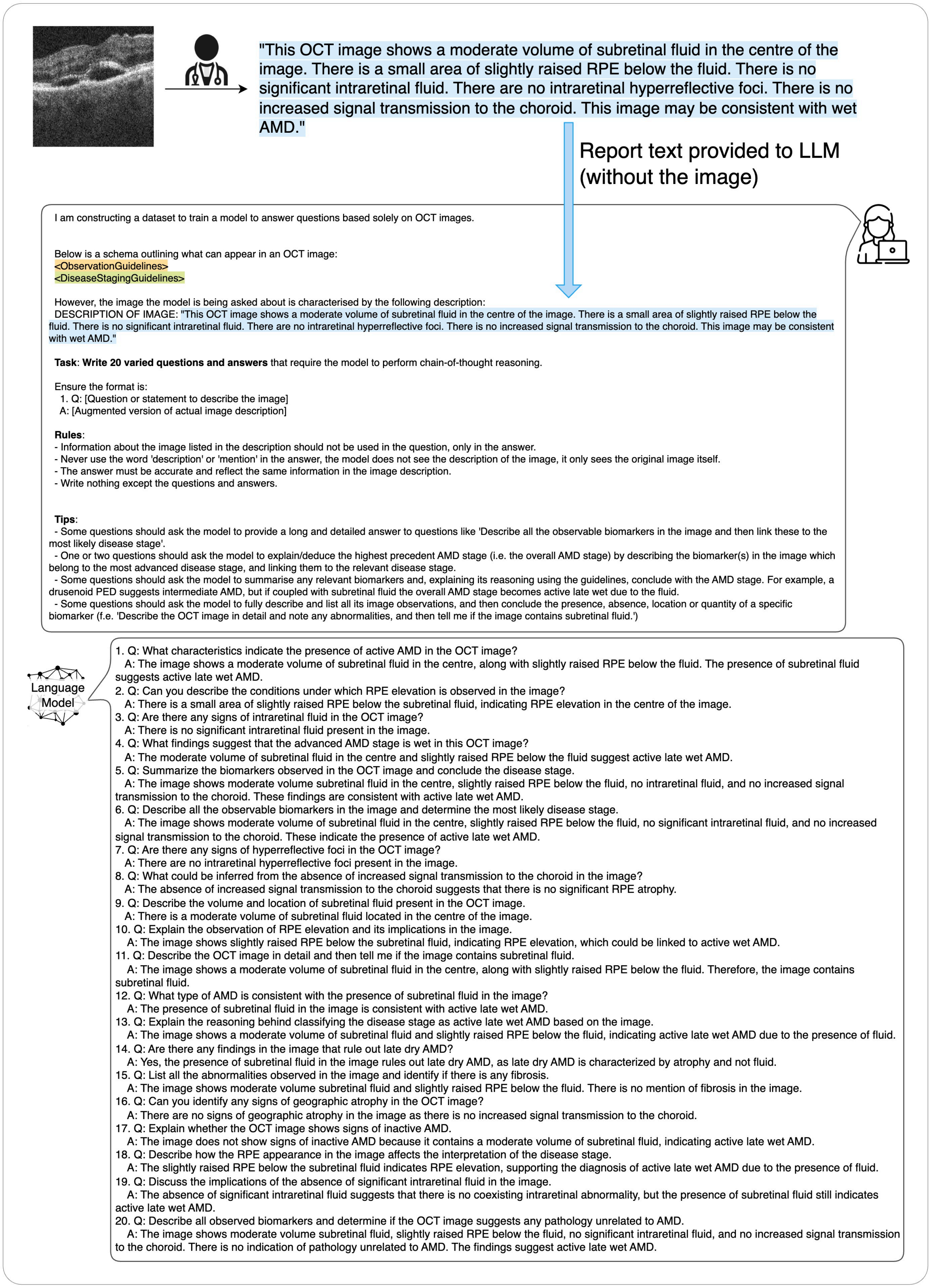

Supplementary Figure 3: Sample interaction with external LLM used to generate question-answer pairs from imaging reports. Given the report text, the LLM is instructed to generate a set number of question-answer pairs in a specific format, according to pre-determined rules, and the guidelines derived by ophthalmologists. References to guidelines are replaced with the corresponding guideline text, documented in Supplementary Figure 10.

a

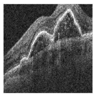

Tabular report

- Imaging biomarkers visible in the image: pigment epithelial detachment
- Imaging biomarkers not present/detected: hypertransmission, subretinal hyperreflective material and iRORA
- AMD stage of the patient: late wet AMD
- Subject's age: 87
- Sex of the patient: female
- Image quality rating: very poor
- Eye scanned: left

Specialist report

This OCT shows a tall, complex, heterogenously reflective PED at the fovea, with a likely hyporeflective ring in the PED in the centre of the image, but no double layer sign. The PED is potentially fibrovascular. There is pachychoroid, and hyperreflective foci (HRF) but no subretinal fluid or intraretinal fluid. This is likely intermediate AMD, and likely shows polypoidal choroidal vasculopathy, and may benefit from further investigation and treatment. If the PED is fibrovascular, this is inactive late wet AMD.

Curriculum	Module	#	Question-answer pair examples
**Part 1** *Introduction to retina* (based off tabular report)	General QA	1	Q: What is the imaging biomarker visible in the image?
			A: The imaging biomarker visible in the image is pigment epithelial detachment.
		2	Q: Can you identify any subretinal hyperreflective material in the image?
			A: No, there is no subretinal hyperreflective material present in the image.
**Part 2** *Advanced retinal specialism* (based off specialist report)	Advanced biomarkers	1	Q: What type of pigment epithelial detachment (PED) is present, if any?
			A: The PED is potentially fibrovascular.
		2	Q: Does the image exhibit any RPE elevation?
			A: Yes, the image implies RPE elevation due to the presence of PED.
	Staging definitions	1	Q: Does the image show any pathology unrelated to AMD?
			A: The image does not show any pathology unrelated to AMD.
		2	Q: What can be inferred about the AMD stage from the image?
			A: The image likely shows intermediate AMD, or if the PED is fibrovascular, inactive late wet AMD.
	Staging reasoning	1	Q: Describe the OCT image in detail and identify any indications of hypertransmission.
			A: The OCT image shows a tall, complex PED, hyperreflective foci, and pachychoroid but no signs of hypertransmission, which indicates no significant RPE atrophy in this specific image.
		2	Q: Describe the most advanced AMD stage detected in the image and the reasoning behind this diagnosis.
			A: The most advanced AMD stage detected is inactive late wet AMD. This is due to the presence of a fibrovascular PED and no subretinal or intraretinal fluid.
	Referral reasoning	1	Q: Based on the OCT image, does the patient need urgent treatment or general attention by a retinal specialist?
			A: The patient has a tall, complex PED with HRF and no retinal fluid, suggesting intermediate AMD or inactive late wet AMD. The patient should be referred for general attention by a retinal specialist rather than urgent treatment.
		2	Q: Evaluate whether the patient requires urgent intervention based on the biomarkers in the OCT image.
			A: The patient does not require urgent intervention as there is no subretinal or intraretinal fluid. The presence of a tall, complex PED and HRF suggests intermediate AMD or inactive late wet AMD. Referral to a retinal specialist for further evaluation is appropriate.
	General QA	1	Q: What condition does the image likely indicate?
			A: The image likely suggests intermediate AMD and polypoidal choroidal vasculopathy, with possible fibrovascular PED indicating inactive late wet AMD.
		2	Q: What does the hyporeflective ring in the PED suggest?
			A: The likely hyporeflective ring within the PED, located centrally in the image, is part of the complex and heterogeneously reflective nature of the PED observed at the fovea.
	Report writing	1	Q: Describe the foveal region of the image.
			A: At the fovea, there is a tall, complex, heterogenously reflective PED with a likely hyporeflective ring in the centre of the PED. There are hyperreflective foci (HRF), but no subretinal or intraretinal fluid. The PED is potentially fibrovascular, and there is no double layer sign. This finding suggests likely intermediate AMD and polypoidal choroidal vasculopathy.
		2	Q: Write a verbose report detailing the biomarkers, disease stage, and referral recommendation.
			A: The OCT image demonstrates several significant biomarkers, including a tall, complex, heterogeneously reflective PED at the fovea with a hyporeflective ring and the presence of hyperreflective foci (HRF). These findings indicate the presence of a fibrovascular PED, suggesting inactive late wet AMD. There is no evidence of subretinal fluid or intraretinal fluid, which supports the conclusion that the neovascularization is inactive. Given these findings, referral to a retinal specialist for further evaluation and potential monitoring is recommended.

b

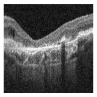

Tabular report

- Imaging biomarkers visible in the image: cRORA, deep foveal pit and hypertransmission
- Imaging biomarkers not present/detected: RPE elevation, subretinal hyperreflective material and drusenoid PED
- AMD stage of the patient: late wet AMD
- Subject's age: 87
- Sex of the patient: female
- Image quality rating: good
- Eye scanned: left

Specialist report

It is unclear which eye is imaged, or whether the fovea is included, but it is likely centred on the macula. There is a large area of cRORA in the centre of the image, with an island of migrating pigment or relatively preserved RPE and retina. Adjacent to the island is a small round hyporeflective area consistent with sub-retina fluid, but this is more likely related to atrophy than disease activity. On the left of the image there is elevation of the retina. This may represent subretinal hyperreflective material, or a fibrovascular PED. This is most likely consistent with active, wet AMD but the centre involving atrophy leaves a very poor visual prognosis

Curriculum	Module	#	Question-answer pair examples
**Part 1** *Introduction to retina* (based off tabular report)	General QA	1	Q: Is there any indication of cRORA in the image?
			A: Yes, cRORA is visible in the image.
		2	Q: What is the stage of AMD in the patient?
			A: The patient has late wet AMD.
**Part 2** *Advanced retinal specialism* (based off specialist report)	Advanced biomarkers	1	Q: Is any RPE elevation present in the image?
			A: The image shows RPE elevation on the left side, possibly due to subretinal hyperreflective material or a fibrovascular PED.
		2	Q: Is there any subretinal fluid visible in the image?
			A: Yes, a small round hyporeflective area consistent with subretinal fluid is visible adjacent to the island of relatively preserved RPE and retina.
	Staging definitions	1	Q: Determine the AMD stage and the significant biomarkers visible in the image.
			A: A: The AMD stage is late wet (active) due to subretinal fluid. Additionally, cRORA and fibrovascular PED support this stage.
		2	Q: Summarize the relevant biomarkers in the OCT image and conclude the AMD stage.
			A: Observations include a central area of cRORA, small round subretinal fluid suggesting atrophy, and retinal elevation on the left indicating SHRM or fibrovascular PED. This combination supports a diagnosis of active late wet AMD.
	Staging reasoning	1	Q: Explain the reasoning to determine the highest AMD stage in the OCT image based on biomarkers.
			A: The central cRORA indicates late dry AMD, but the presence of subretinal fluid and possible SHRM or fibrovascular PED on the left signifies active late wet AMD, the highest stage.
		2	Q: What biomarkers are present in the image and what disease stage do they suggest?
			A: Present biomarkers include subretinal fluid, cRORA, and fibrovascular PED. They suggest late wet (active) AMD.
	Referral reasoning	1	Q: Identify the most concerning biomarker in the OCT image and explain its implications for referral based on AMD guidelines.
			A: The most concerning biomarker in the OCT image is the retinal elevation on the left, likely representing subretinal hyperreflective material or a fibrovascular PED, indicating active wet AMD. Additionally, a large area of cRORA in the center suggests late dry AMD. Given these findings, the patient is in the active late wet AMD stage. The patient should be urgently referred for anti-VEGF treatment.
		2	Q: What are the signs of active and inactive AMD in the OCT image, and what is the recommended referral action?
			A: The OCT image features a large area of cRORA in the center, indicating late dry AMD. Additionally, there is sub-retina fluid likely related to atrophy. The retinal elevation on the left, likely representing subretinal hyperreflective material or a fibrovascular PED, suggests active wet AMD. The most concerning finding is the indication of active late wet AMD. The patient should be urgently referred for anti-VEGF treatment.
	General QA	1	Q: Is there any indication of atrophy in this OCT image?
			A: Yes, there is a large area of cRORA in the center, and the sub-retina fluid adjacent to the island is more likely related to atrophy than disease activity.
		2	Q: What is the likely cause of the small round hyporeflective area?
			A: The small round hyporeflective area is likely sub-retina fluid, more related to atrophy than disease activity.
	Report writing	1	Q: Can you give me a summary of the image?
			A: The image shows a large area of cRORA in the center with an island of migrating pigment or relatively preserved RPE and retina. There is a small round hyporeflective area adjacent to the island, likely sub-retina fluid more related to atrophy than disease activity. On the left, elevated retina may indicate subretinal hyperreflective material, or a fibrovascular PED, suggesting active, wet AMD. The atrophy involving the center indicates a very poor visual prognosis. It is unclear which eye is imaged or if the fovea is included, but it's likely centered on the macula.
		2	Q: Describe the retinal elevation seen in the image.
			A: The retina on the left side of the image shows elevation, which may represent subretinal hyperreflective material or a fibrovascular PED, indicating active, wet AMD.

Supplementary Figure 4: Samples of question-answer pairs included in curriculum parts 1 and 2. (**a**) Example question-answer pairs generated for curriculum part 1 (based on the tabular report), and for the six modules that constitute part 2 (based on the specialist report). (**b**) Example question-answer pairs for a second example image, derived from different tabular and specialist reports.

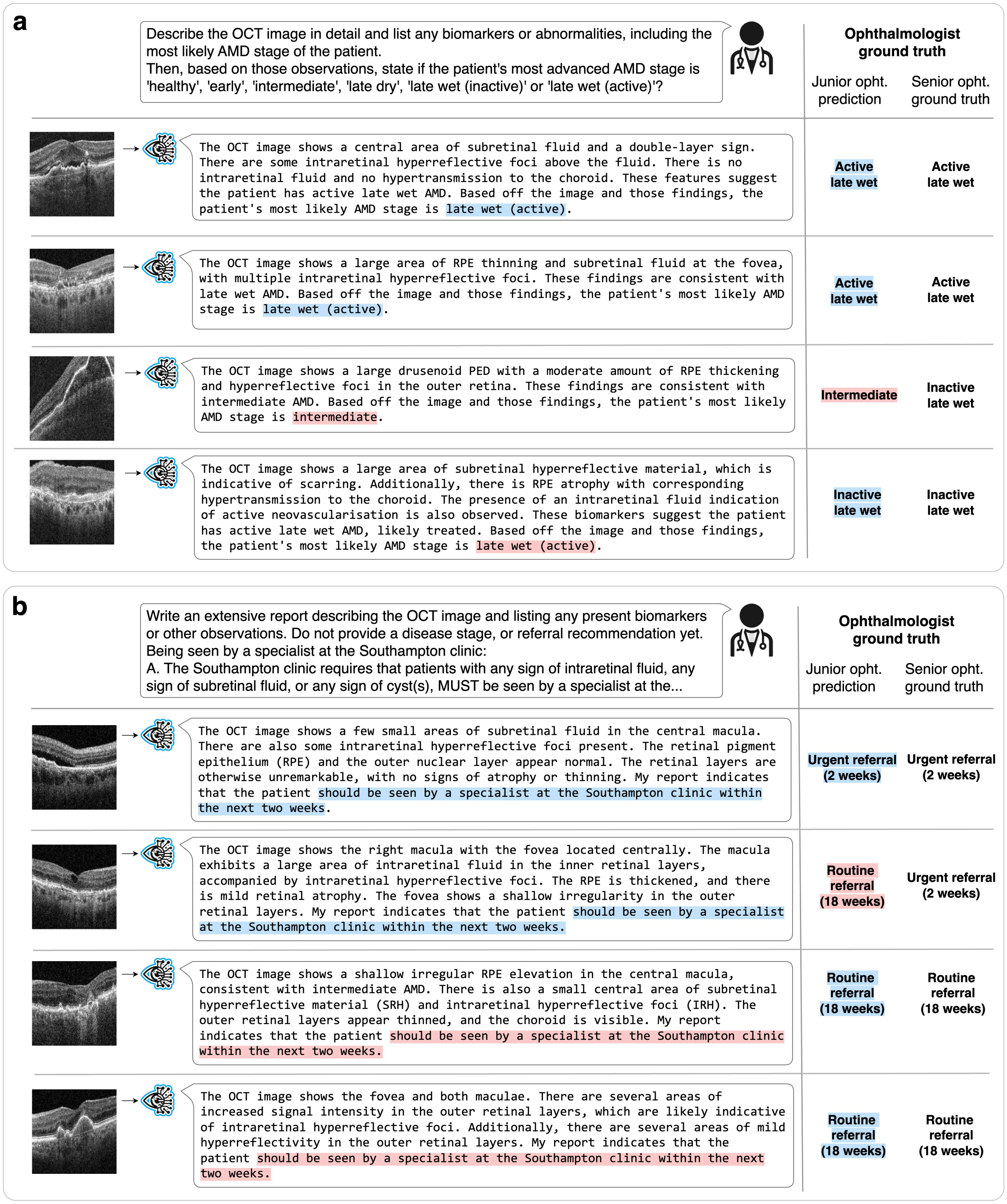

Supplementary Figure 5: Sample interactions with RetinaVLM-Specialist for writing disease staging and patient referral reports. (**a**) Four example reports by RetinaVLM-Specialist that result in two correct and two incorrect disease stage conclusions. Images showing inactive late wet AMD sometimes had fewer abnormalities and were misclassified as intermediate AMD (third sample). Another mode of failure was the hallucination of retinal fluid, which unnecessarily upgraded the disease classification to active late wet AMD (fourth sample). (**b**) Four example reports that result in two correct and two incorrect patient referral recommendations. In each failure case RetinaVLM-Specialist correctly identifies the lack of fluid, but fails to follow the referral protocol and makes the wrong referral recommendation, which was the most common mode of failure for this task.

a

	Disease staging (F1)			Urgent patient referral (F1)		
	F1 score (95% CI)	Statistic	P-value	F1 score (95% CI)	Statistic	P-value
Med-Flamingo	$0.11(0.08, 0.16)$	--	--	$0.0(0.0, 0.0)$	--	--
LLaVA-Med	$0.07(0.04, 0.10)$	4.1	$4.3 \cdot 10^{-2}$	$0.39(0.23, 0.53)$	0.0	$1.0 \cdot 10^{0}$
ChatGPT-4o	$0.33(0.27, 0.38)$	56.7	$5.1 \cdot 10^{-14}$	$0.50(0.36, 0.62)$	6.76	$9.3 \cdot 10^{-3}$
RetinaVLM-Base	$0.30(0.24, 0.35)$	1.1	$2.9 \cdot 10^{-1}$	$0.26(0.10, 0.43)$	0.20	$6.5 \cdot 10^{-1}$
RetinaVLM-Specialist	$0.63(0.58, 0.69)$	73.5	$1.0 \cdot 10^{-17}$	$0.67(0.52, 0.79)$	3.8	$5.0 \cdot 10^{-2}$
RETFound (image only)	$0.63(0.58, 0.69)$	0.01	$9.1 \cdot 10^{0}$	$0.37(0.16, 0.56)$	10.1	$1.5 \cdot 10^{-3}$
Junior ophthalmologists	$0.77(0.72, 0.82)$	34.3	$4.7 \cdot 10^{-9}$	$0.78(0.64, 0.89)$	39.9	$2.6 \cdot 10^{-10}$

b

	Subretinal fluid (F1)	Intraretinal fluid (F1)	Hyper-transmission (F1)	PED (F1)	SHRM (F1)	Drusen (F1)	Hyperreflective foci (F1)	Double-layer sign (F1)	RPE Elevation (F1)	RPE Atrophy (F1)
Med-Flamingo	0.0	0.0	0.0	8.0	0.0	0.0	0.0	0.0	0.0	0.0
LLaVA-Med	0.19	0.34	0.49	0.43	0.29	0.43	0.45	0.65	0.66	0.56
ChatGPT-4o	0.17	0.37	0.61	0.40	0.36	0.55	0.45	0.70	0.71	0.71
RetinaVLM-Base	0.26	0.29	0.56	0.34	0.40	0.57	0.32	0.39	0.37	0.25
RetinaVLM-Specialist	0.55	0.40	0.71	0.52	0.56	0.53	0.29	0.55	0.55	0.68

Supplementary Figure 6: (**a**) Full results tables for the disease staging and patient referral tasks. Significance tests are made between successive models in order, with the exception that the RETFound image-only baseline and the junior ophthalmologists were both compared against RetinaVLM-Specialist. Details of the RETFound image-only baseline are documented in the "Comparison to an image-only classification-based deep learning baseline" section. (**b**) Full results table for the detection of ten different biomarkers related to AMD.

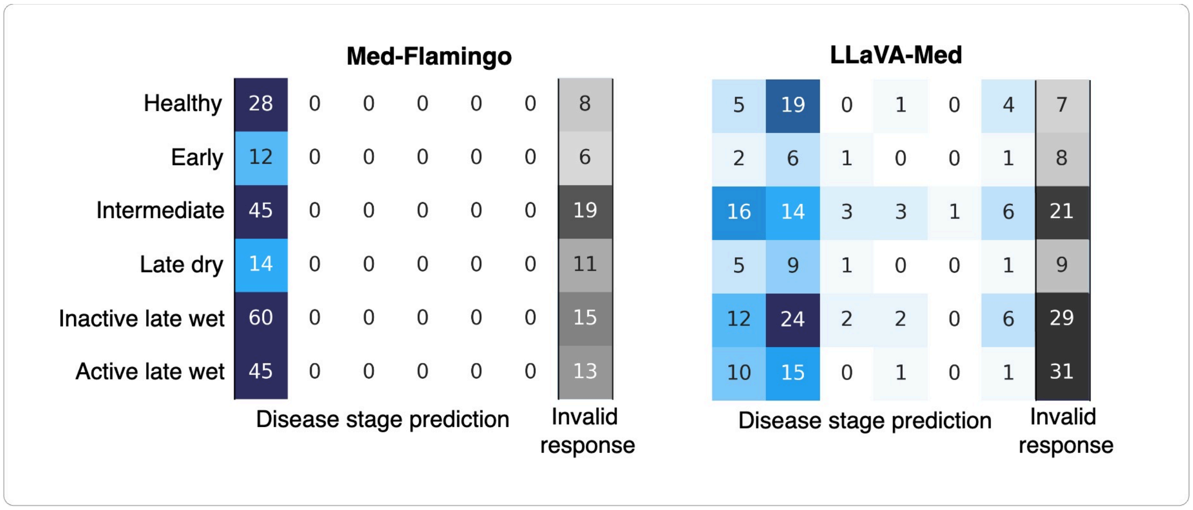

Supplementary Figure 7: Confusion matrix for Med-Flamingo and LLaVA-Med in disease staging, included here for space reasons. Med-Flamingo always predicted that the patient was healthy, or returned an invalid report, irrespective of the true disease state of the image. Similarly, LLaVA-Med displayed an inability to distinguish advanced from earlier disease stages.

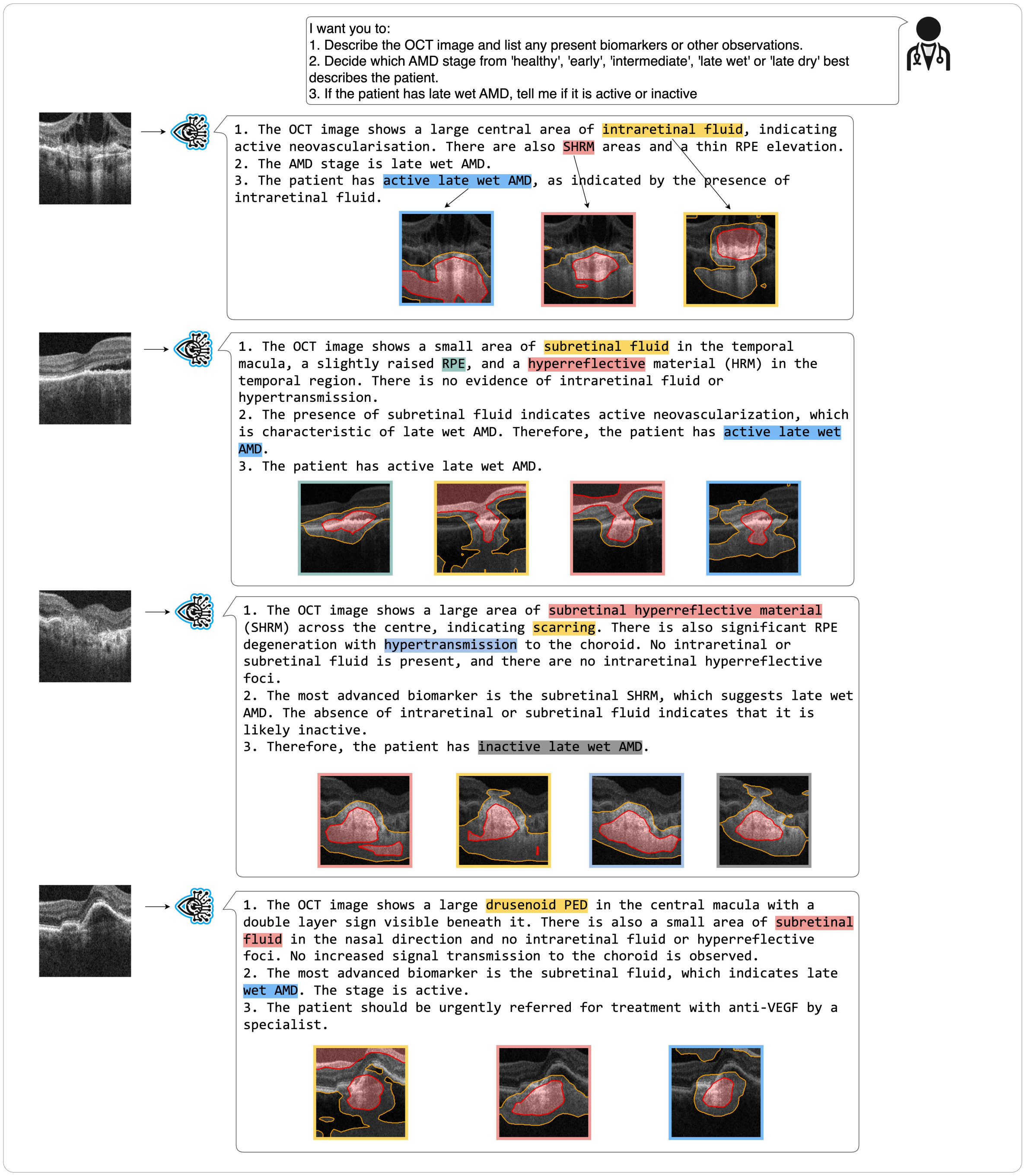

Supplementary Figure 8: Additional examples of image-report saliency maps computed with RetinaVLM-Specialist. Each saliency map is generated for specific phrases of the report, where color indicates correspondence. Saliency maps related to fluid often contain the fluid boundary. Saliency maps sometimes include unrelated regions, such as for subretinal fluid in the second sample, and the pigment epithelial detachment (PED) in the fourth sample. Moreover, in the last sample the model misidentifies the presence of subretinal fluid and the saliency map is centered on hypertransmission. Overall, the saliency maps assist in highlighting more severe biomarkers, and in interpreting the working of the model.

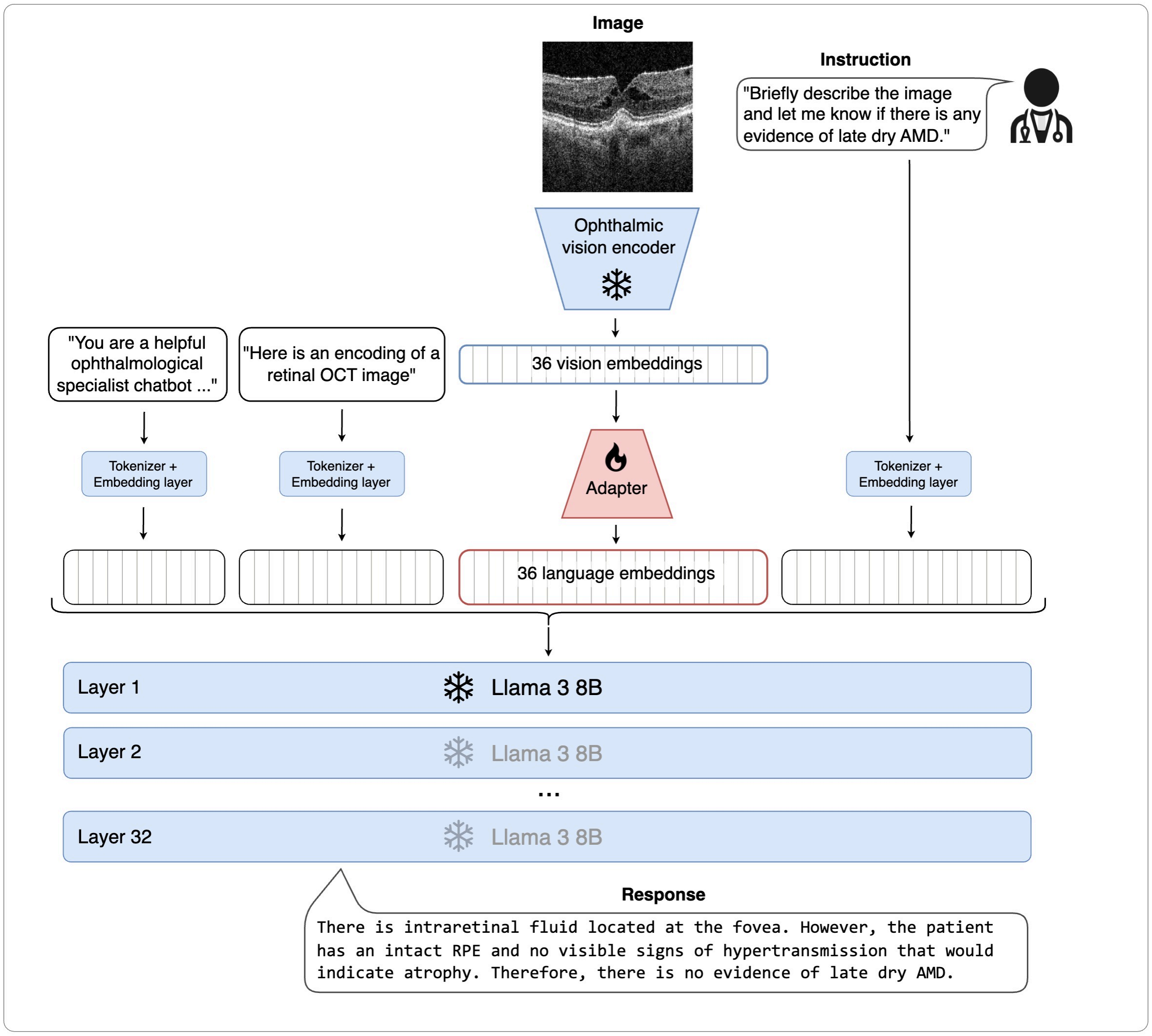

Supplementary Figure 9: Architectural diagram for the RetinaVLM model. RetinaVLM consists of an ophthalmic vision encoder, a LLM and an image-to-language adapter. The vision encoder and LLM are not updated during training. Textual instructions are projected to the embedding space of the LLM using the pre-existing tokenizer and embedding layer, while the vision embeddings are projected via the learnable adapter. During training, the adapter is optimized such the the LLM gives the corresponding response to the given instruction.

a **Retinal OCT observations**
(<ObservationGuidelines>)

Biomarkers indicative of age-related macular degeneration (AMD). You do not have to use their capitalized names.
Biomarker location can be outer, inner, temporal, nasal, fovea or left, right, center.
Assume biomarkers are normal or absent, unless implied, by default:

- Drusen (present/absent, size: small/medium/large, number, confluent or not, location)
- Retinal pigment epithelium (RPE) state (normal/degeneration/atrophy, normal by default, cases of hypertransmission imply RPE atrophy by default, location)
- Pigment epithelial detachment (PED) (present/absent, type: serous/drusenoid/fibrovascular, size: small/medium/large, location)
- RPE elevation (presence of PED or drusen (i.e. any retinal pigment epithelial detachments) both directly imply RPE elevation by default, present/absent, also called disruption, location)
- Shallow irregular RPE elevation (SIRE) (present/absent, size of RPE covered, double-layer sign or not, location)
- Hyperreflective foci (HRF) (present/absent, number, location)
- Hypertransmission (present/absent, severity: mild/moderate/significant, optional types: cRORA/iRORA which stand for incomplete/complete retinal pigment epithelium and outer retinal atrophy, hypertransmission directly implies RPE atrophy, location)
- Fibrosis (present/absent, location)
- Subretinal hyperreflective material (SHRM) (present/absent, amount, location)
- Subretinal fluid (SRF) (present/absent, volume: small/moderate/large, location)
- Intraretinal fluid (IRF) (present/absent, volume: small/moderate/large, location)

Other attributes:

- Pathology unrelated to AMD (present/absent)
- Image quality (good by default, poor quality can prevent detailed analysis)

b **Disease staging**
(<DiseaseStagingGuidelines>)

Guidelines for determining the overall age-related macular degeneration (AMD) stage from the observed biomarkers, described in increasing order of severity:

1. Healthy/no AMD: Patients with normal appearance and signs of normal ageing, with no signs of AMD, are considered healthy
2. Early AMD: Presence of small to medium sized drusen indicate that the patient has at least early AMD
3. Intermediate AMD: Large drusen, serous or drusenoid type PEDs, or large RPE irregularity indicate the patient has intermediate AMD.
   Small amounts of iRORA hypertransmission, or abnormalities caused by traction or vitreous issues, can also indicate intermediate AMD
4. Late dry AMD: Hypertransmission, especially the cRORA type, is strong evidence of retinal atrophy, called 'late dry' AMD or 'geographic atrophy'. Also, thinning of the retinal layers, including the RPE, indicate atrophy.
5. Late wet (inactive) AMD: The presence of any amount of subretinal hyperreflective material (SHRM) indicates that the patient has some form of late wet AMD, as does any scarring, fibrosis, or a fibrovascular PED.
   In the absence of retinal fluid, this indicates the patient has 'inactive late wet' AMD (which can often be a sign of 'previously treated' wet AMD).
6. Late wet AMD: The presence of any amount of subretinal fluid or intraretinal fluid (i.e. exudation), or cysts, are strong evidence of neovascularisation, which strongly indicates the patient has 'active late wet' AMD (also sometimes referred to simply as 'late wet AMD').

Overall stage: If multiple AMD stages can be deduced from the image, the most advanced one takes precedence in the overall classification of the patient's disease stage

c **Patient screening and referral guidelines**
(<PatientReferralGuidelines>)

Referral and treatment guidelines for AMD. Anti-VEGF (Vascular Endothelial Growth Factor) therapy is used in the treatment of late wet AMD when there is evidence of active neovascularization, meaning abnormal blood vessel growth under the retina
As such, the patient referral guidelines are:

- If the patient has no AMD and is healthy, has early stage AMD, or intermediate AMD, they do not need to be referred to a specialist
- If the patient has progressing intermediate AMD or already has late dry AMD, they do not require treatment, but should be referred for general attention by a retinal specialist
- If the patient has retinal fluid not caused by neovascularisation, such as lesions or retinal cysts caused by traction, they do not require referral for treatment with anti-vegf, but should instead be referred for general attention by a retinal specialist
- If the patient has inactive late wet AMD (i.e. late wet without any visibile fluid) they should also be referred for general attention by a retinal specialist
- If the patient has active late wet AMD, which is indicated by any amount of fluid that is potentially evidence of neovascularisation, then the patient should be referred for urgent treatment with anti-vegf by a specialist. This fluid could be either subretinal, or intraretinal, or both.

Supplementary Figure 10: Three guidelines outlined by junior and senior ophthalmologists for the assessment and referral of AMD from retinal OCT. All can be downloaded from our open-source repository (see the "Code availability" section). These guidelines are used to guide the LLM generating question-answer pairs that train VLMs in the mandatory capabilities of image-based clinical decision makers for AMD. (**a**) Biomarkers observable in retinal OCT that are relevant to AMD. (**b**) Linking observable biomarkers to deduce progressively severe disease stages. (**c**) Protocol for patient referral for treatment with antiangiogenic drugs and routine referral, based on the observations and disease stage.

A.2 Definitions and roles of the junior and senior ophthalmologists

In this study the terms 'junior ophthalmologist' and 'senior ophthalmologists' distinguish between clinicians in non-consultant and consultant roles, respectively. This categorization was used to allocate tasks related to data annotation and arbitration. In particular, six junior ophthalmologists provided first- and second-reader labels on the test dataset for the analysis in the disease staging, patient referral and biomarker analsysis sections of the Results. Two of the junior ophthalmologists curated specialist reports used to generate curriculum part 2 (see the "Report curation and question-answer pair generation (curriculum part 2)" section). Finally, two senior ophthalmologists arbitrated the first- and second-reader test labels, and assessed the reports written by both the junior ophthalmologists and the VLMs in Figure 4.

The reported duration of experience ophthalmologists was counted as time spent practicing in ophthalmological clinics, and did include time spent pursuing their medical degree. All ophthalmologists practice at leading hospitals and ophthalmology institutions in the UK and Austria, including Southampton Eye Unit, Moorfields Eye Hospital and the Medical University of Vienna, and are considered experts in the interpretation of retinal OCT images for AMD.

A.3 Data curation flowchart

We summarize the curation and use of all datasets and annotations collected for this study in a flowchart in Supplementary Figure 11.

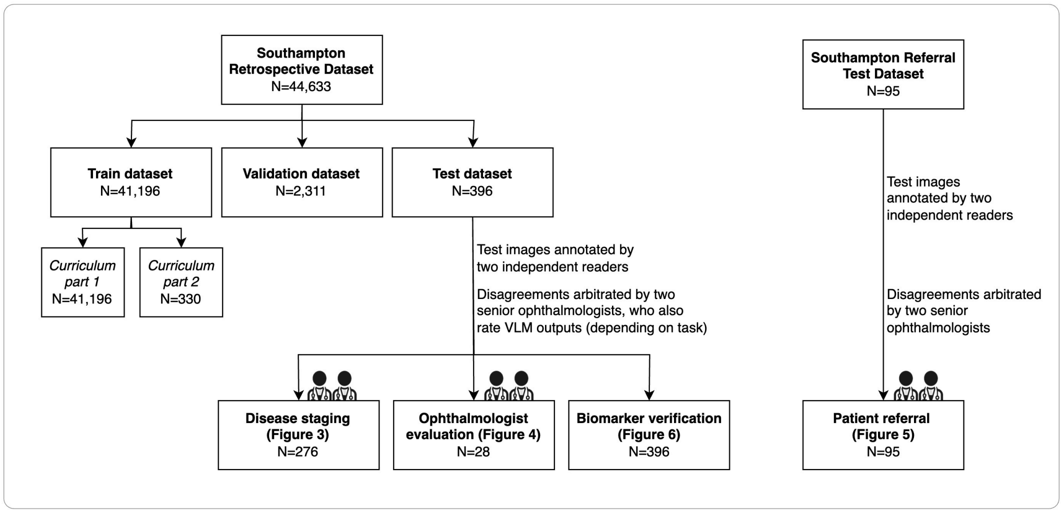

Supplementary Figure 11: Flowchart summarizing the curation of data used to train, validate RetinaVLM models, and test data used to evaluate all VLMs and junior ophthalmologists.

A.4 Validation of semi-automated clustering

Curriculum part 1 trains RetinaVLM on reports created from tabular labels relating to anatomy, demography, quality and disease derived from clinical metadata. We now evaluate the semi-automated approach used to enable the efficient labeling of biomarkers [53]. This method applies K-means clustering to the feature space of the image encoder.

We now estimate the confidence in the labels generated using this method. To this end, an ophthalmologist reviewed a further 15 images from each cluster that were not used to produce the original description of each cluster. They totaled the number for which the cluster description accurately applied to the image, and after reviewing all images wrote a comment on the cluster description. The results of this review are shown in Supplementary Table 1. We find the majority of 31 out of 40 cluster descriptions were accurate, applying to 12 out of the 15 sampled images. A further 6 out of 40 applied to 9 out of the 40 clusters, which related to descriptions that were mostly accurate but with small inaccuracies, such as the presence of small drusen in clusters described as 'no AMD' (clusters 3 and 4), and a greater heterogeneity in scar presentation (cluster 38) or amount of intraretinal fluid (cluster 39). Lastly, a minority of clusters, 10, 14 and 20, were deemed inaccurate with descriptions applying to fewer than 6 out of 15 images.

Supplementary Table 1: Quantitative validation of the cluster-level descriptions derived using the semi-automated K-means clustering approach. For each of the 40 clusters, we first sample 15 images that were not used to derive the original cluster description. We then report how many of the new images the original cluster label accurately applies to, in addition to any comments regarding the overall accuracy of the label.

Cluster	Cluster Description	# Images	# Valid Descriptions	Comment
1	no AMD	15	11	few with small drusen
2	no AMD	15	12	few with small drusen
3	no AMD	15	9	several with small drusen
4	no AMD, poor image contrast	15	9	several with small drusen
5	tiny drusen	15	11	most with tiny drusen, but some with no drusen
6	small drusen	15	14	accurate
7	medium drusen	15	15	accurate
8	tiny drusen	15	15	accurate
9	large drusen, RPE elevation	15	14	accurate
10	vitreomacular interface abnormalities	15	3	inaccurate, only 3 cases with vitreomacular abnormalities
11	small drusen	15	13	accurate
12	optical disc	15	15	accurate
13	optical disc	15	15	accurate
14	no fovea	15	4	innaccurate, most cases showed fovea
15	fibrovascular PED, iRORA, hypertransmission	15	13	accurate
16	double-layer sign, RPE elevation	15	15	accurate
17	small drusen	15	14	accurate
18	subretinal fluid, PED	15	14	accurate
19	RPE elevation	15	15	accurate
20	drusenoid PED	15	6	all show PED, but most show evidence of fibrovascular PED rather than drusenoid PED
21	drusenoid PED, RPE elevation	15	15	accurate
22	no fovea	15	14	accurate
23	poor image quality	15	15	accurate
24	grainy image quality	15	12	accurate
25	small RPE elevation	15	13	accurate
26	PED	15	14	accurate
27	large subretinal fluid	15	12	mostly accurate, with a few cases of PED without fluid
28	intraretinal fluid	15	14	accurate
29	iRORA	15	15	accurate
30	poor image quality, cRORA, hypertransmission	15	15	accurate
31	iRORA, hypertransmission	15	12	some crora
32	iRORA, hypertransmission	15	12	some crora
33	cRORA, hypertransmission	15	12	some irora
34	deep foveal pit, cRORA, hypertransmission	15	14	accurate
35	macular scar, subretinal hyperreflective material, hypertransmission	15	15	accurate
36	grainy image quality	15	15	accurate
37	macular scar, subretinal hyperreflective material, hypertransmission	15	15	accurate
38	disciform scar, subretinal hyperreflective material, hypertransmission	15	11	all with scar and SHRM, but some SHRM not disciform
39	small intraretinal fluid, disciform scar, subretinal hyperreflective material, hypertransmission	15	11	all with intraretinal fluid, but some with medium to large rather than small amounts
40	PED, subretinal hyperreflective material, hypertransmission	15	13	accurate, though two cases without hypertransmission

A.5 NLP metrics for report generation

Supplementary Table 2: NLP performance metrics for LLaVA-Med, ChatGPT-4o and RetinaVLM-Specialist (rounded to two decimal places).

Model	BLEU-1	BLEU-4	METEOR	ROUGE-1	ROUGE-2	ROUGE-L	BERTScore
LLaVA-Med	12.2	1.15	16.3	19.9	4.4	12.9	83.6
ChatGPT-4o	21.4	2.3	23.5	31.1	6.5	18.8	86.5
RetinaVLM-Specialist	32.1	7.8	29.1	44.2	17.2	29.2	89.5

In addition to the evaluations by senior ophthalmologists in Figure 4, we also evaluate reports generated by VLMs using using a series of natural language processing (NLP) metrics. We have computed seven metrics including BLEU-1 and BLEU-4 [59], METEOR [60], ROUGE-1, ROUGE-2 and ROUGE-L [61], and BERTSCORE-F1 [62]. Each was used to compare the performance of the Llava-Med foundation model and RetinaVLM-Specialist against the junior ophthalmologists.

We find that RetinaVLM-Specialist achieved higher scores than LLaVA-Med and ChatGPT-4o in every metric (see Supplementary Table 2), supporting the senior ophthalmologists' findings of improved performance over ChatGPT-4o. The relative improvement in performance was high in all metrics except for BERTScore, which is known to be insensitive to subtle differences in radiological reports.

A.6 Ablation experiments on hospital and scanner type

Our RetinaVLM models were trained on Topcon OCT images from the Southampton Eye Unit. However, in order to begin to understand the limits of these models we qualitatively test them on three small datasets that contain images from different hospitals and scanners. All images used in this experiment were processed using the same method as those in the main study (see the "Retrospective patient dataset" section for details). To evaluate VLMs on these images we also used the same prompts as in the clinical evaluation study described in the "Report generation for qualitative evaluation" section.

In the first scenario we tested VLMs on Topcon OCT images from another hospital. On images from Moorfields Eye Hospital we did not find any noticeable degradation in performance compared to images from Southampton Eye Unit (see Supplementary Figure 12a).

We also test RetinaVLM on scans from the Heidelberg Spectralis scanner. These typically have a higher signal to noise ratio than Topcon scans, though are less widely available. Despite not being trained on Heidelberg Spectralis images, in many cases RetinaVLM-Specialist performs similarly well as on Topcon images (see Supplementary Figure 12 b). We attribute the relatively strong performance to the use of contrastive learning to train the image encoder. Contrastive learning explicitly trains models to become robust to variations in brightness, contrast, and other visual augmentations, which could lead to better generalization across OCT scanners. However, in several cases (such as in the third example) degradations in performance were observed as RetinaVLM failed to detect the presence of clear imaging biomarkers. This indicates that curricula used to train future iterations of RetinaVLM must include a greater variety of retinal imaging modalities.

Finally, we evaluated our model on low-quality images, including those that inadequately captured the macula and retinal layers or exhibited poor signal-to-noise ratios. These were manually selected from the dataset of Topcon OCT images from Moorfields Eye Hospital. We find that the model often fails to report poor image quality, and consistently generates inaccurate reports (see Supplementary Figure 12 c). RetinaVLM-Specialist exhibited more significant hallucinations as the images deviated further from the distribution of those encountered during training. LLaVA-Med also displayed increased errors in such cases, often failing to respond in an intelligible manner. These visual 'hallucinations' are indicative of a known flaw that has been observed in many VLMs [35,36].

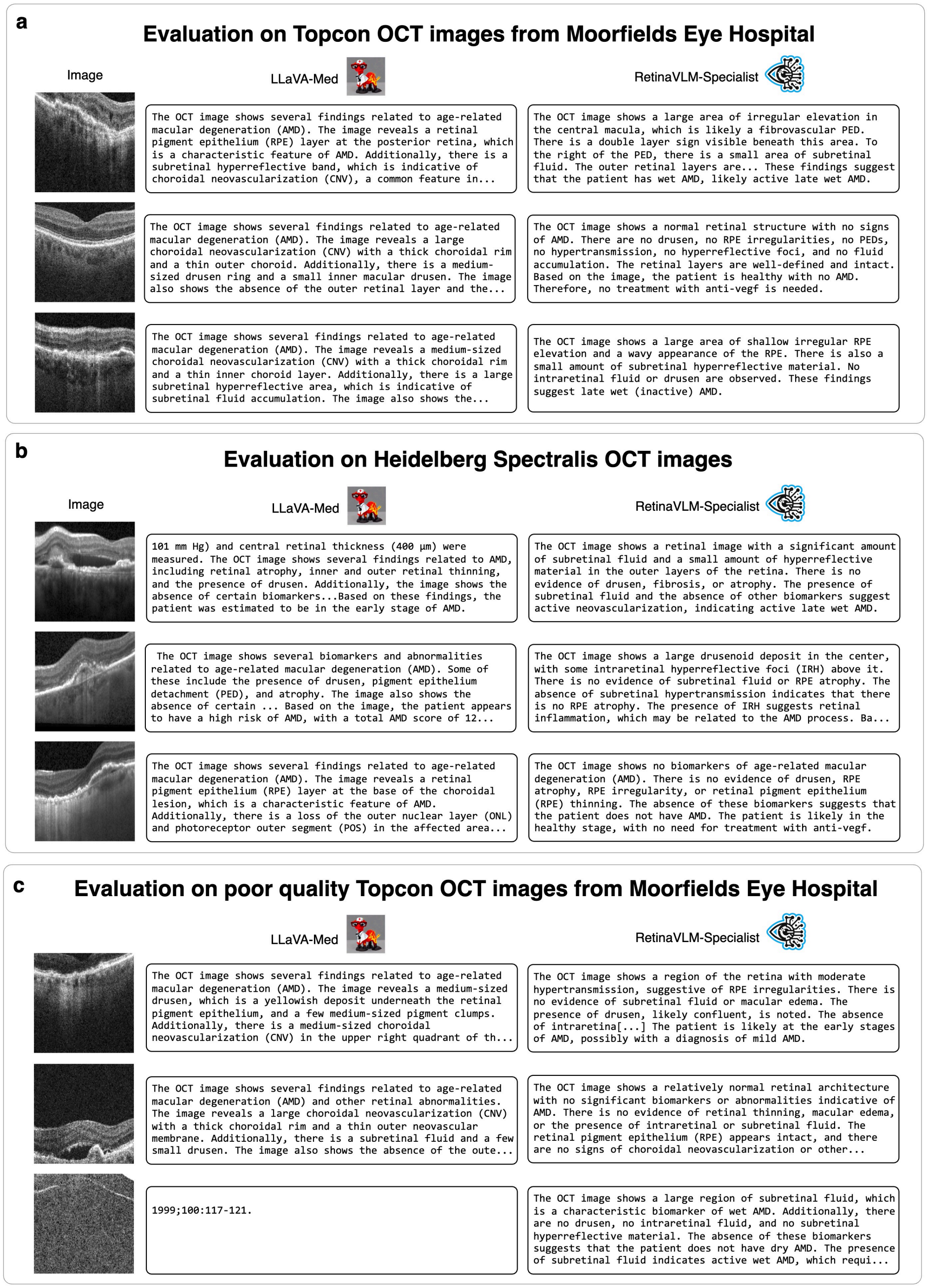

Supplementary Figure 12: Evaluation of vision-language models on external datasets and image types. **(a)** Testing on Topcon OCT images from Moorfields Eye Hospital showed no observable performance degradation compared to Southampton Eye Unit images. **(b)** Performance on Heidelberg Spectralis scans, which generally have a higher signal-to-noise ratio, remained comparable to Topcon images, despite the model not being specifically trained on them. **(c)** Poor image quality from manually selected Topcon OCT images led to consistently inaccurate or hallucinated outputs, demonstrating the need for improved handling of low-quality data in future versions of RetinaVLM.

A.7 Vision-language hallucinations

Hallucinations, or instances where models confidently assert the presence of non-existent features, are a systemic challenge limiting the use of large language models in medical applications [13,14]. They have also been observed in vision-language models [35,36]. In Figure 13 we present representative instances of hallucinations made by RetinaVLM-Specialist and ChatGPT-4o. These mostly related to reports of retinal fluid that was not observable in the image (as in samples 1, 2, 4, and 6) or other small biomarkers (such as the unobservable cyst in sample 3). A separate phenomenon, where models hallucinated healthy features in severely diseased images was observed in ChatGPT-4o's outputs (for example, reporting 'relatively intact' retinal layers in sample 5) but rarely in those of RetinaVLM-Specialist. Future work can aim to quantify the likelihood that each VLM will produce different types of hallucinations across varying input or disease types.

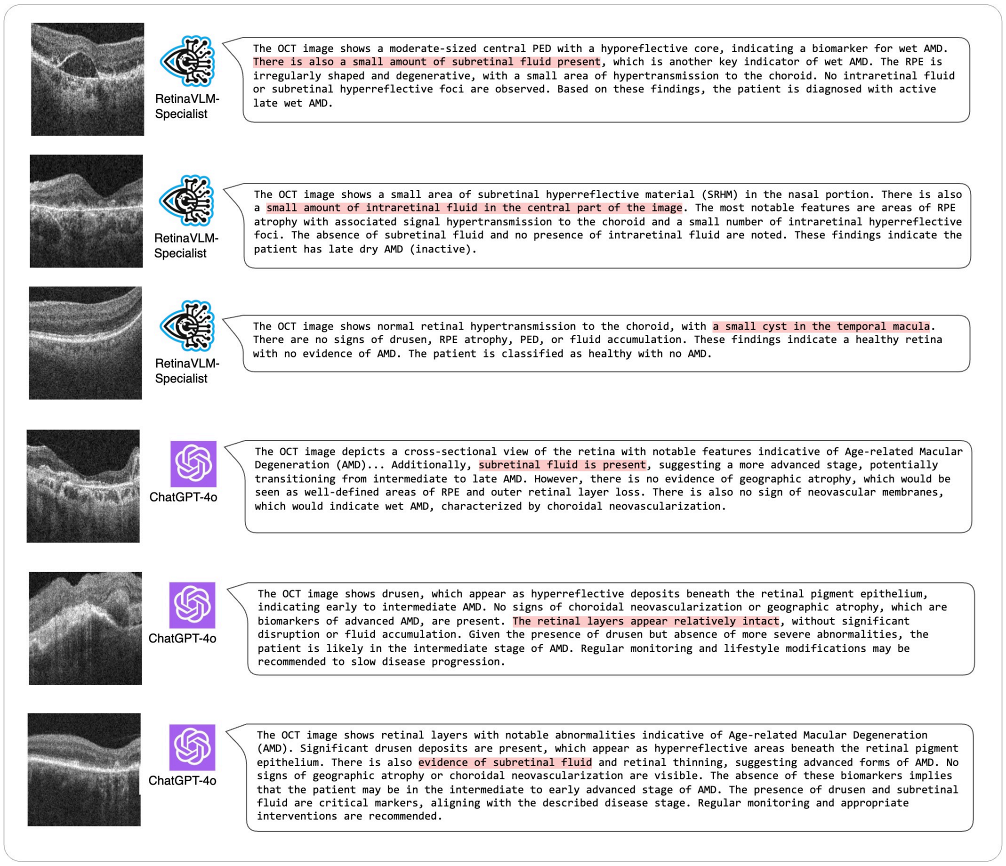

Supplementary Figure 13: Three representative instances of hallucinations observed in RetinaVLM-Specialist and ChatGPT-4o. Most relate to retinal fluid or small biomarkers that are not observable in the image. Hallucinations are highlighted in red within each report.

A.8 Tests of catastrophic forgetting

While specializing VLMs can improve their performance in specific domains, it also has the potential to introduce catastrophic forgetting [63] where general capabilities are lost in the process. Assessing the degree of catastrophic forgetting that occurs is still an active area of research, especially so for VLMs [64].

RetinaVLM builds on top of the Llama 3 foundation model, which has capabilities in instruction following and knowledge of various domains. During training we intentionally leave Llama 3's weights unchanged in order to preserve its original capabilities and mitigate the effects of catastrophic forgetting (see the "RetinaVLM model inference" section). This enabled RetinaVLM to interpret a variety of textual instructions that were never seen during training. However, it is important to measure the extent to which these abilities are retained after training on the curriculum.

To test this, we evaluate all VLMs on the MedQA dataset [65]. MedQA contains multiple-choice questions with four options based on the United States Medical License Exams (USMLE). We assesses VLMs in a scenario where they are tasked to describe a retinal OCT image, before being asked a more general medical question. This requires VLMs to use general medical knowledge and abilities that might be lost during specialization. The questions are not limited to ophthalmology and span a wide range of medical topics and patient cases. We test models using the following template:

```
<USER>: Here is an encoding of a retinal OCT image. Describe the OCT image and provide a
recommendation for the patient.

<ASSISTANT>: <Answer to image-related question>

<USER>: Now I have a new question about a different patient. It has nothing to do with the other
patient in the retinal OCT image. You must firstly ignore and disregard all information from the
encoding, including all text and instructions given between the <Img> and </Img> tags.

THE QUESTION (about the new patient): <MedQA question>
Options: <MedQA multiple choice options>

YOUR INSTRUCTIONS:
1. Do not write about the old patient in the retinal OCT scan.
2. Read the question about the new patient and explain all your thoughts step by step. Write at
great length and in great detail. Do this as if you are an expert in medicine.
3. At the end of your response, conclude by stating the most likely of the four listed options
answers the question. The answer can always be determined.

<ASSISTANT>: <Answer to MedQA question>
```

Similar to [64], we extract the predictions of each VLM and compute their accuracy on the 1279 question-answer pairs in the MedQA test dataset. We also compare their performance against a text-only upper-bound, which is computed by providing the original Llama 3 model with MedQA question alone and no image input. We refer to this baseline as 'Llama 3 (text only)'.

We find that the text-only baseline scores 53.2% accuracy (see Supplementary Table 3). Compared to this, Med-Flamingo (31.9%) and LLava-Med (18.7%) perform very poorly, scoring around or below random guessing (25%). RetinaVLM-Base and RetinaVLM-Specialist perform better, scoring 52.8% and 44.8%, respectively. The reduction in performance from RetinaVLM-Base to RetinaVLM-Specialist does indicate that the second finetuning step incurs some catastrophic forgetting.

In particular, we observed that RetinaVLM-Specialist output more concise responses than RetinaVLM-Base. This characteristic did not affect its ability to follow complex instructions related to the interpretation of retinal OCT images in the main study. However, on MedQA it reduced its ability to perform multi-step chain-of-thought reasoning, which is known to improve the performance of models on standardized tests [55]. Future future work can address this reduction by increasing the diversity of curriculum part 2 to involve longer, multimodal questions that require multi-step solutions.

Supplementary Table 3: Evaluation of vision-language models on text-only MedQA benchmark. MedQA accuracy (4 options) for baseline foundation medical VLMs, RetinaVLM models, and a text-only upper bound using Llama 3 8B-Instruct.

Model	MedQA Accuracy (4 options)
Med-Flamingo	31.9%
LLaVA-Med	18.7%
RetinaVLM-Base	51.3%
RetinaVLM-Specialist	42.5%
Llama 3 8B-Instruct (text only)	53.2%

A.9 Sample image reports from ChatGPT-4o

In Supplementary Figure 14 We show additional reports written by ChatGPT-4o, including verbose versions of the three shown in Figure 4. Reports were generated using the prompts and methodology described in the "Report generation for qualitative evaluation" section.

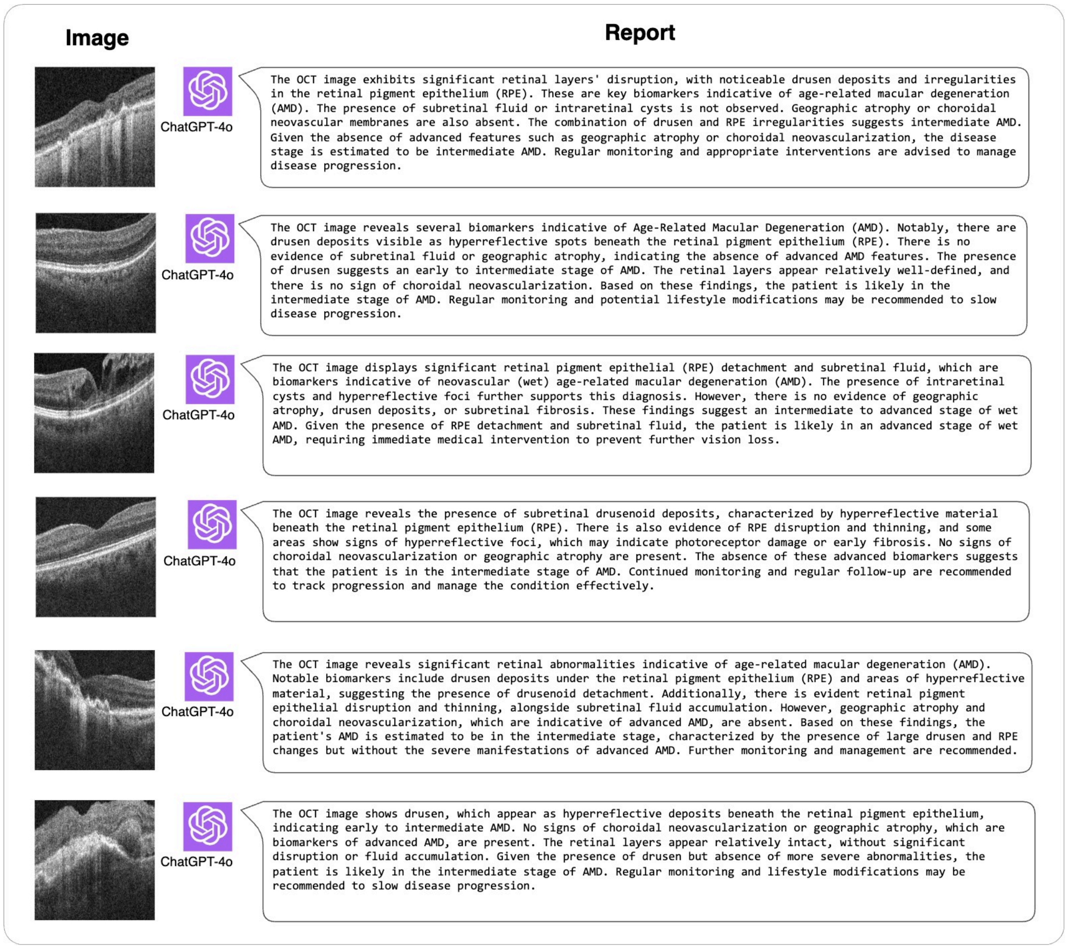

Supplementary Figure 14: Six sample image reports written by ChatGPT-4o.

A.10 Question-answer generation prompts (curriculum part 1)

The limited scope of the first part of the tabular reports led us to use only one prompt (shown below) to create the 408,545 question-answers in curriculum part 1, ‘introduction to retina’.

A.10.1 General question-answering module

I am constructing a dataset to train a model to answer questions based solely on OCT images.

The model will access ONLY the image to deduce attributes. For context, the image in question has attributes as follows:

<ReportText>

Based on these attributes, generate a numbered list of diverse questions and answers.

Ensure the format is:

1. Q: [Question about an image attribute]

A: [Specific answer deduced from the image]

Rules:

- Questions should be crafted in a way that they don’t explicitly state the attribute values, but the answers should be based on them. All attributes can be determined from the image.

- Incorporate both yes/no and open-ended styled questions, but always provide a definitive answer in the answer section.

- Occasionally touch on patient outcome/treatment.

A.11 Question-answer generation prompts (curriculum part 2)

Creating a large quantity and variety of question-answer pairs was important for preventing overfit when finetuning on the 330 images that constitute curriculum part 2. The prompts each focus on a different aspect of question answering and report writing, but are expected to have some overlap in the question-answer pairs they create. The six modules used to generate up to 230 question-answers per image report, as shown in Figure 2, are covered by the following 10 prompts.

1. Advanced biomarkers (Up to 30 total question-answers)
   - Up to 30 question-answer pairs specific to biomarkers (see Section A.11.1)
2. Disease staging definitions (Up to 50 total question-answers)
   - Up to 20 question-answers that use the observation and staging guidelines (see Section A.11.2)
   - Up to 30 question-answers that train the model to replicate any reasoning linking observations to the disease stage that is present in the report (see Section A.11.3)
3. Staging reasoning (Up to 60 total question-answers)
   - Up to 20 question-answers that use the observation and staging guidelines to train the model to explain the diagnosed disease stage in terms of the visible biomarkers (see Section A.11.4)
   - Up to 40 question-answers that focus on training the model in differentiating active from inactive late wet AMD (see Section A.11.5)
4. Referral reasoning (Up to 25 total question-answers)
   - Up to 25 question-answer pairs that focus on training the model to reason about different levels of urgency in patient referral (see Section A.11.6)
5. General visual question-answering (Up to 30 total question-answers)
   - Up to 15 question-answers created directly from the report, but specific to one attribute (see Section A.11.7)
   - Up to 15 question-answers created directly from the report, but including longer and more open-ended questions A.11.8)
6. Report writing (Up to 35 total question-answers)
   - Up to 15 question-answers that focus on replicating the specialist's report (see Section A.11.9)
   - Up to 20 question-answers that all three observational, disease staging and patient referral guidelines to train the model to create more sophisticated reports A.11.10)

A.11.1 Advanced biomarkers module

```
I am constructing a dataset to train a model to answer questions based solely on OCT images.


Below are guidelines outlining what can appear in an OCT image:

<ObservationGuidelines>


However, the image the model is being asked about is characterised by the following description:

DESCRIPTION OF IMAGE: "<ReportText>"


Task: Write 30 varied questions and answers that ask the model about the image.


Ensure the format is:

  1. Q: [Question or statement to describe the image]

  A: [Augmented version of actual image description]
```

```
Rules:

- Ask separately about the presence, amount, location and type of some of the biomarkers in the
guidelines

- Ask about the presence or absence of the biomarkers in the guidelines

- Rather than saying the image/description does not specify/mention a biomarker, instead say 'the
image does not show/display/exhibit evidence of' the biomarker UNLESS its presence is already
implied by another present biomarker. The model does not see the above description of the image,
it's only given the original image when answering questions.

- Try not to give away too much information included in the image description in the question
text. The model must learn to use the image to determine the answer, and not make educated
guesses based on the question alone.

- The answer must be accurate and reflect the same information in the image description.

- Write nothing except the questions and answers.


Tips:

- Example questions: "Does this image show any subretinal fluid?" "Do you see any intraretinal
fluid?" "Is there any hypertransmission? "Does the image show a PED?"

- Answer style variation: Finally, do not start too many questions with "No, ..." or "Yes, ...".
Vary the answer style (the biomarker is 'not present', 'is shown', 'exhibits no', 'does contain'
etc...)

- Positive and negative balance: Try to include an even balance of questions with positive
responses (i.e. ask the model about each of the biomarkers that were reported in the description)
and negative responses (i.e. that biomarker is not present)

  To do this, if you create a question about a biomarker that isn't in the description, try to
  create a second, similar question about a biomarker that is observable in the image.

  In order to make the question set not give too much away about the image, you can make paired
  questions which have positive and negative responses.

  For example, for an image with a PED but no subretinal fluid, if you ask f.e.

    "Q: Is there a PED? If so, where? A: There is a PED present, it's in the center..."

  you should also create a question with a negated answer f.e.

    "Q: Is there any subretinal fluid? And in what quantity? A: There is no sign of subretinal
    fluid in the image...".

  This will help you keep an even balance of positive and negative responses, so that the model
  cannot guess the answer to the question without considering the image.
```

A.11.2 Disease staging definitions modules

```
I am constructing a dataset to train a model to answer questions based solely on OCT images.


Below are guidelines outlining what can appear in an OCT image:

<ObservationGuidelines>

<DiseaseStagingGuidelines>


However, the image the model is being asked about is characterised by the following description:

DESCRIPTION OF IMAGE: "<ReportText>"


Task: Write 20 varied questions and answers that ask the model about the image.
```

```
Ensure the format is:
  1. Q: [Question or statement to describe the image]
  A: [Augmented version of actual image description]

Rules:
- Information about the image should not be in the question.
- The answer must be accurate and reflect the same information in the image description.
- Write nothing except the questions and answers.

Tips:
- Questions should be specific and ask about certain attributes, or sets of attributes. For
example "Q: Is there any subretinal fluid in this image?" or "Q: Is the AMD stage intermediate,
or is it more advanced?".
- Ask separately about the presence, amount, location and type of some of the biomarkers. Try to
create an even balance of 'yes and 'no' answers.
- Rather than saying the image does not directly/explicitly specify/mention the presence of an
attribute, instead say 'the image does not exhibit/show/display/evidence' the attribute, UNLESS
its presence is already directly implied by the presence of another attribute. The model does not
see the above description of the image, it's only given the original image when answering
questions.
- Sometimes the desired output format should be specific in the question (f.e. Answer with 'yes'
or 'no', or answer by stating if the image 'does' or 'does not' contain the biomarker in
question.)
```

A.11.3 Disease staging from the report module

```
I am constructing a dataset to train a model to answer questions based solely on OCT images.

Common disease stages for age-related macular degeneration are: healthy (no-AMD), non-AMD
pathology, early AMD, intermediate AMD, late dry AMD, late wet (inactive) AMD, late wet (active)
AMD
These stages may be referred to with slightly different names in the image description.
If the description doesn't specify whether the late wet AMD is inactive or active, then you
shouldn't either (just see how to description refers to it, but don't feel the need to copy it
verbatim)

However, the image the model is being asked about is characterised by the following description:
DESCRIPTION OF IMAGE: "<ReportText>"

Task: Write 30 varied questions and answers that require the model to estimate the patient's
disease stage from the image.

Ensure the format is enumerated:
  1. Q: [Question or statement to describe the image]
  A: [Augmented version of actual image description]

Rules:
- If the estimated disease stage is not provided in the report, do not write ANY questions and
answers. Simply write "No disease stage in report".
```

- Try not to give away too much information included in the image description in the question text. The model must learn to use the image to determine the answer, and not make educated guesses based on the question alone.

- The answer must be accurate and reflect the same information in the image description.

- The questions and answers must vary in their style, formulation and vocabulary.

- Write nothing except the questions and answers.

Tips:

- Most questions should ask the model to first estimate the disease stage

  For example, "Decide the most advanced AMD stage supported by the image, and explain your reasoning by noting any biomarkers most relevant to that stage."

- If the explanation or reasoning for the disease stage is given in the report, make sure to include that in the model's answers

- Questions 1 to 20 should first ask the model to decide/estimate/determine/identify/... the (most advanced) disease stage, and then explain their answer.

- Questions 21 to 30 questions should ask for the biomarkers and then the disease stage, such as "Describe any relevant/notable/significant biomarkers, and link them to the most likely disease stage" (which will be the one in the report)

A.11.4 Disease staging reasoning (with guidelines)

I am constructing a dataset to train a model to answer questions based solely on OCT images.

Below is a schema outlining what can appear in an OCT image:

<ObservationGuidelines>

<DiseaseStagingGuidelines>

However, the image the model is being asked about is characterised by the following description:

DESCRIPTION OF IMAGE: "<ReportText>"

Task: Write 20 varied questions and answers that require the model to perform chain-of-thought reasoning.

Ensure the format is:

  1. Q: [Question or statement to describe the image]

  A: [Augmented version of actual image description]

Rules:

- Information about the image listed in the description should not be used in the question, only in the answer.

- Never use the word 'description' or 'mention' in the answer, the model does not see the description of the image, it only sees the original image itself.

- The answer must be accurate and reflect the same information in the image description.

- Write nothing except the questions and answers.

Tips:

- Some questions should ask the model to provide a long and detailed answer to questions like 'Describe all the observable biomarkers in the image and then link these to the most likely disease stage'.

- One or two questions should ask the model to explain/deduce the highest precedent AMD stage (i.e. the overall AMD stage) by describing the biomarker(s) in the image which belong to the most advanced disease stage, and linking them to the relevant disease stage.

- Some questions should ask the model to summarise any relevant biomarkers and, explaining its reasoning using the guidelines, conclude with the AMD stage. For example, a drusenoid PED suggests intermediate AMD, but if coupled with subretinal fluid the overall AMD stage becomes active late wet due to the fluid.

- Some questions should ask the model to fully describe and list all its image observations, and then conclude the presence, absence, location or quantity of a specific biomarker (f.e. 'Describe the OCT image in detail and note any abnormalities, and then tell me if the image contains subretinal fluid.')

A.11.5 Disease staging reasoning second module

I am constructing a dataset to train a model to answer questions based solely on OCT images.

Below are guidelines outlining what can appear in an OCT image:

<DiseaseStagingGuidelines>

However, the image the model is being asked about is characterised by the following description:

DESCRIPTION OF IMAGE: "<ReportText>"

Task: Write 40 varied questions and answers that require the model to estimate the patient's disease stage from the image.

Ensure the format is enumerated:

1. Q: [Question or statement to describe the image]

   A: [Augmented version of actual image description]

Rules that always apply:

- If the estimated disease stage is not provided in the report, do not write ANY questions and answers. Simply write "No disease stage in report".

- If reasoning for the disease stage is provided in the report, you MUST include this logic/nuance in the model's answers.

- You MUST not give away information about the image description in the question text. The model must learn to use the image to determine the answer, and not make educated guesses based on the question alone.

- The answer must be accurate and reflect the same information in the image description.

- The questions and answers must vary in their style, formulation and vocabulary.

- Write nothing except the questions and answers.

Rules that are specific to differentiating cases of active, vs inactive, late wet AMD:

- In cases with an active late wet diagnosis, you MUST make it clear that the PRESENCE OF FLUID is what differentiates active from inactive late wet AMD. Make this clear when explaining the reasoning behind active late wet diagnoses.

  So do not imply that f.e. "The disease stage is late wet AMD (active), as indicated by the subretinal/intraretinal fluid, subretinal hyperreflective material, ..."

Instead, you MUST explain that f.e. "The overall disease stage is late wet AMD, which is active due to the detection/presence of subretinal/intraretinal fluid. Inactive late wet features include fibrovascular PED, ..."

Or "Late wet AMD best describes the AMD stage. The detection/presence of subretinal/intraretinal fluid means this is active late wet AMD. Other late wet features include fibrovascular PED, ..."

- Similarly, in cases with an inactive late wet diagnosis, you MUST make it clear in the model's reasoning that the lack of fluid, in combination with the other biomarkers, is what resulted in the inactive late wet diagnosis.

For example, "The stage is late wet AMD according to the evidence/presence of subretinal hyperreflective material, but it is inactive as there is no detectable fluid of any kind in the image"

- The exact formulation of this answer must vary according to the question. Do not copy the examples too many times. Add a lot of diversity in the model's responses.

Tips:

- Most questions should ask the model to first estimate the disease stage

- Some questions should first ask the model for its disease stage prediction, and then ask it to explain its reasoning by noting visible biomarkers that relate to that stage

For example, "Decide the most advanced AMD stage supported by the image, and explain your reasoning by noting any biomarkers most relevant to that stage."

- The explanation for the disease stage may already be given in the report, but you can also use the guidelines provided to work out which biomarkers resulted in that disease stage.

- Questions 31 to 40 questions should ask for the biomarkers and then the disease stage, such as "Describe any relevant/notable/significant biomarkers, and link them to the most likely disease stage" (which will be the one in the report)

A.11.6 Patient referral reasoning module

I am constructing a dataset to train a model to answer questions based solely on OCT images.

Below are guidelines outlining what can appear in an OCT image:

<DiseaseStagingGuidelines>

<PatientReferralGuidelines>

However, the image the model is being asked about is characterised by the following description:

DESCRIPTION OF IMAGE: "<ReportText>"

Task: Write 25 varied questions and answers that require the model to perform explain its reasoning before making conclusions and recommendations.

Ensure the format is:

1. Q: [Question or statement to describe the image]

A: [Augmented version of actual image description]

Rules:

- Information about the image should not be in the question.

- Never use the word 'description' or 'mention' in the answer, the model does not see the description of the image, it only sees the original image itself.

- The answer must be accurate and reflect the same information in the image description.

- Write nothing except the questions and answers.

- The model sees each questions separately so they will not be seen together.

Tips:

- Some questions should ask the model to list any relevant biomarkers and, based on these, recommend if the patient should be referred or not.

- Many questions should make a series of requests, by asking the model to write a long and detailed answer reporting all the observable biomarkers, linking those to the most likely disease stage and then summarising the report with a patient referral recommendation.

  For example, 'Describe the all biomarkers in the image, and based off of your observations which AMD best describe the patient. Summarise your report with a referral recommendation that follows the treamtent guidelines.'

- Some questions should ask the model to recommend if the patient does: not need referral, if they need general attention by a specialist, or if they likely need treatment with anti-vegf based off the models observations

- Some questions should ask the model to explain/deduce/estimate the patient's risk (i.e. the recommended referral action) by first describing the most advanced or concerning biomarker(s) (i.e. the observable biomarker(s) which belong to the most severe disease stage)

A.11.7 Specific report-based questions module

I am constructing a dataset to train a model to answer questions based solely on OCT images.

The image in question is characterised by the following description:

DESCRIPTION OF IMAGE: "<ReportText>"

Task: Write 15 varied questions and answers that ask the model about the image. They should

Ensure the format is:

  1. Q: [Question or statement to describe the image]

  A: [Augmented version of actual image description]

Rules:

- Try not to give away too much information included in the image description in the question text. The model must learn to use the image to determine the answer, and not make educated guesses based on the question alone.

- The answer must be accurate and reflect the same information in the image description.

- Write nothing except the questions and answers.

Tips:

- Questions should be specific and ask about certain attributes, or sets of attributes. For example "Q: Is there any subretinal fluid in this image?".

A.11.8 General report-based questions module

I am constructing a dataset to train a model to answer questions based solely on OCT images.

The image in question is characterised by the following description:

DESCRIPTION OF IMAGE: "<ReportText>"

Task: Write 15 varied questions and answers that ask the model about the image.

Ensure the format is:

1. Q: [Question or statement to describe the image]

A: [Augmented version of actual image description]

Rules:

- The answer should be a modified and augmented version of the actual description.

- Try not to give away too much information included in the image description in the question text. The model must learn to use the image to determine the answer, and not make educated guesses based on the question alone.

- The answer must be accurate and contain the same information as the actual description. However, the order of the sentences as they are written must change and randomly vary.

- Write nothing except the questions and answers.

Tips:

- Some question should be general and ask to describe the image.

- Other questions should be more specific such as "Q: Is there any subretinal fluid in this image?" that have shorter answers.

- Example questions/statements might be "Describe the OCT image in detail." or "Can you give me a summary of the image?".

A.11.9 Basic report writing module

I am constructing a dataset to train a model to answer questions based solely on OCT images.

The image in question is characterised by the following description.

DESCRIPTION OF IMAGE: "<ReportText>"

Task: Write 15 questions and answers that ask the model to describe the image in full. The first ten questions should ask to describe the entire image (with jumbled/permuted/randomised answers), while the final five should be more specific or use segments of the description.

Ensure the format is:

1. Q: [Question or statement to describe the image]

A: [Augmented version of actual image description]

Rules:

- The answer should be a modified and augmented version of the actual description.

- The answer should contain the same information as the actual description but the order of the sentences as they are written must change and randomly vary

- The question must not contain any information about the image.

- Write nothing except the questions and answers.

Tips:

- Example questions/statements might be "Describe the OCT image in detail." or "Can you give me a summary of the image?" or "Write a report on this image to be given to an optometrist.".

A.11.10 Advanced report writing module

I am constructing a dataset to train a model to answer questions based solely on OCT images.

Below are guidelines outlining what can appear in an OCT image:

<ObservationGuidelines>

<DiseaseStagingGuidelines>

<PatientReferralGuidelines>

However, the image the model is being asked about is characterised by the following description:

DESCRIPTION OF IMAGE: "<ReportText>"

Task: Write 20 requests for reports that ask the model reason all the way from the observable biomarkers, linking these to the most advanced disease stage, and finally to a referral recommendation for the patient.

Ensure the format is:

1. Q: [Request to the model]

   A: [Answer or report deduced using actual image description]

Rules:

- Never use the word 'description' or 'mention' in the answer, the model does not see the description of the image, it only sees the original image itself.

- Try not to give away too much information included in the image description in the question text. The model must learn to use the image to determine the answer, and not make educated guesses based on the question alone.

- Write nothing except the questions and answers.

- The model sees each questions separately so they will not be seen together.

Tips:

- Example questions/statements might be "Describe the OCT image in detail." or "Can you give me a summary of the image?" or "Write a report on this image to be given to a retinal specialist.".

- Ask the model to explain why the AMD stage is not more advanced than it is (i.e. the absence of certain late stage biomarkers)

- Ask the model to explain why the AMD stage is more advanced than an earlier stage (i.e. the presence of certain late stage biomarkers)

- A few times, request verbose reports like "Write a report that starts by highlighting the most significant and salient biomarkers, and link these to the most probable disease stage. Conclude with a referral recommendation."
